# Global prevalence of coronavirus disease 2019 reinfection: a systematic review and meta-analysis

**DOI:** 10.1186/s12889-023-15626-7

**Published:** 2023-04-28

**Authors:** Joyeuse Ukwishaka, Yves Ndayishimiye, Esmeralda Destine, Celestin Danwang, Fati Kirakoya-Samadoulougou

**Affiliations:** 1 Maternal Child and Community Health Division, Rwanda Bio-Medical Center, Kigali, Rwanda; 2 IntraHealth International, Kigali, Rwanda; 3 Centre de Recherche en Epidémiologie, Biostatistique et Recherche Clinique, Ecole de Santé Publique, Université Libre de Bruxelles, Brussels, Belgium; 4 Clinton Health Access Initiative, Inc., Boston, MA USA

**Keywords:** COVID-19, Reinfection, Prevalence

## Abstract

**Background:**

In December 2019, severe acute respiratory syndrome coronavirus 2 (SARS-CoV-2) emerged with a high transmissibility rate and resulted in numerous negative impacts on global life. Preventive measures such as face masks, social distancing, and vaccination helped control the pandemic. Nonetheless, the emergence of SARS-CoV-2 variants, such as Omega and Delta, as well as coronavirus disease 2019 (COVID-19) reinfection, raise additional concerns. Therefore, this study aimed to determine the overall prevalence of reinfection on global and regional scales.

**Methods:**

A systematic search was conducted across three databases, PubMed, Scopus, and ProQuest Central, including all articles pertaining to COVID-19 reinfection without language restriction. After critical appraisal and qualitative synthesis of the identified relevant articles, a meta-analysis considering random effects was used to pool the studies.

**Results:**

We included 52 studies conducted between 2019 and 2022, with a total sample size of 3,623,655 patients. The overall prevalence of COVID-19 reinfection was 4.2% (95% confidence interval [CI]: 3.7–4.8%; *n* = 52), with high heterogeneity between studies. Africa had the highest prevalence of 4.7% (95% CI: 1.9–7.5%; *n* = 3), whereas Oceania and America had lower estimates of 0.3% (95% CI: 0.2–0.4%; *n* = 1) and 1% (95% CI: 0.8–1.3%; *n* = 7), respectively. The prevalence of reinfection in Europe and Asia was 1.2% (95% CI: 0.8–1.5%; *n* = 8) and 3.8% (95% CI: 3.4–4.3%; *n* = 43), respectively. Studies that used a combined type of specimen had the highest prevalence of 7.6% (95% CI: 5.8–9.5%; *n* = 15) compared with those that used oropharyngeal or nasopharyngeal swabs only that had lower estimates of 6.7% (95% CI: 4.8–8.5%; *n* = 8), and 3.4% (95% CI: 2.8–4.0%; *n* = 12) respectively.

**Conclusion:**

COVID-19 reinfection occurs with varying prevalence worldwide, with the highest occurring in Africa. Therefore, preventive measures, including vaccination, should be emphasized to ensure control of the pandemic.

**Supplementary Information:**

The online version contains supplementary material available at 10.1186/s12889-023-15626-7.

## Background

Coronavirus disease 2019 (COVID-19) is an infectious disease caused by severe acute respiratory syndrome coronavirus 2 (SARS-CoV-2), also known as the 2019 novel coronavirus (2019-nCoV) [1, 2]. It emerged in December 2019 with a very high transmissibility rate. The first case was reported in Wuhan City, Hubei Province, China [1]. The World Health Organization (WHO) declared it a pandemic on March 11, 2020 [1, 3].

Before the 2019 pandemic, other epidemics of coronaviruses have been reported, including SARS-CoV-1 and Middle East Respiratory Syndrome coronavirus (MERS-CoV), which have a high fatality rate compared with that of SARS-CoV-2 [4–8]. These infections were rapidly controlled before they became a global emergency [9–13]. Approximately 80% of SARS-CoV-2 infections are asymptomatic [14]. Human-to-human transmission occurs by respiratory droplets, close contact, and possibly aerosol and fecal–oral contact [14–16].

Globally, more than 650 million COVID-19 cases and 6.6 million deaths have been reported [17]. Europe has reported over 41.3% of all global cases, followed by the Americas (28.4%), Western Pacific (15.9%), Southeast Asia (9.3%), East Mediterranean (3.6%), and Africa (1.4%) [17].

The basic reproduction number of COVID-19 is estimated to be between 1.4 and 2.4, with an average incubation period of 4–5 days, while the recovery rate is 98.8% [17–19]. However, severity and recovery depend on various factors in which an increased risk is found in older individuals or those with underlying conditions such as cancer, diabetes mellitus, cardiovascular diseases, and chronic respiratory diseases [20–25].

SARS-CoV-2 continues to change over time, and some variants have raised global health concerns because they are associated with increased risks of transmission, clinical worsening of the disease, or resistance to containment measures [26, 27]. Therefore, they are collectively named variants of concern (VOC) [26]. Among these variants, we cite Alpha, Beta, Gamma, Delta, and Omicron, the recently circulating VOC [26, 28]. The Omicron variant has the capacity to skip acquired immunity from prior infection, hence increasing the risk of reinfection [29–32]. Other factors that increase COVID-19 reinfection include female sex, older age, underlying comorbidity, unvaccinated status, and being a healthcare provider [33–36]. Compared with that of the initial COVID-19 infection, the risk of case fatality in reinfected cases decreased by 68% [33].

Various efforts have been made to control this pandemic, such as vaccines, social distancing, wearing of face mask, hand hygiene, and isolation of infected patients [37–42]. Despite these efforts, some cases of reinfection have been reported since the first wave of COVID-19 [43–45].

To date, there is no conventional definition of COVID-19 reinfection. European countries consider a range of 45–90 days from a previously confirmed infection to a newly confirmed infection, whereas the European Center for Disease Control (ECDC) proposes a period of more than 60 days after the first infection [46]. The American Center for Disease Control (CDC) recommends that reinfection be considered after a sufficient period has elapsed for immunity to mount up [47].

Different other authors consider reinfection when SARS-CoV-2 is detected 90 days after the initial or prior infection, whereas before that period, it is usually considered as relapse, reactivation, or re-positivity of the initial SARS-CoV-2 infection [48–51]. Dafna et al. defined re-positivity, reinfection, and relapse based on both clinical and epidemiological aspects [51]. They clinically defined reinfection as a recurrence of clinical symptoms and a positive polymerase chain reaction (PCR) after 90 days of the previous infection or within 90 days if there was a symptom—free period and two recorded negative PCR [51].

In addition to the lack of a conventional definition of reinfection, evidence of the occurrence of SARS-CoV-2 reinfection is still limited. This systematic review and meta-analysis determined the current prevalence of COVID-19 reinfection at global and regional levels, considering a longer follow-up period.

## Methods

### Study design

Systematic review and meta-analysis were reported in accordance with the Preferred Reporting Items for Systematic Reviews and Meta-Analyses (PRISMA).

### Search strategy

A digital search was conducted on June 14, 2022, using three databases: PUBMED, SCOPUS, and ProQuest Central. The search strategy used for each database is shown in the (Additional file 1: Table S1). The search was restricted to studies published from 2019 (when the first case of COVID-19 occurred). We considered all observational studies that reported sufficient data to compute the prevalence of COVID-19 reinfection without language, age, or sex restriction. Case reports and series were excluded because they could not provide the denominator for the calculation of prevalence. For this study, COVID-19 reinfection was defined as the development of a new COVID-19 infection after a previous infection that was declared cured. The references of the included articles were scrutinized as potential sources for additional studies.

### Data management and study selection

The titles and abstracts of the relevant articles retrieved from the databases were transferred to the Rayyan online software, which was used to organize the literature search results. After the removal of duplicates, the selection of articles based on title and abstract was performed independently by three reviewers (UJ, YN, and DE), with disagreements being addressed through discussions to reach a consensus. Subsequently, the final inclusion of articles was decided based on the full texts independently assessed by the three authors.

### Data extraction and quality assessment

From relevant eligible articles, the following data were extracted: author name, year of publication, country (where the study was conducted), study design, type of used data, definition of reinfection, total number of participants with primary infection, number of reinfected patients, population of the study, type of specimen, vaccination status considerations, and mean/median age of the study participants. The outcome was that people previously infected with SARS-CoV-2 subsequently developed a new infection after being declared as cured.

The National Institutes of Health (NIH) quality assessment tool for cohort and cross-sectional studies was used to evaluate the methodological quality and risk of bias of the included articles. The 14 items of the NIH tool were used independently by three authors, UJ, NY, and ED, for the evaluation of every study, and a consensus was reached through discussions. Each item was scored as 0 if the condition was not met or 1 if the condition was met. Articles with a final overall score less than or equal to 4 were categorized as being of poor quality (high risk of bias), 5–10 as being of fair quality (medium risk of bias), and 11 and above as being of good quality (low risk of bias). More details on each question can be found in the Additional file 1 and online [52].

### Data analysis

We used STATA/SE software version 17.0 for data analysis. Random-effects meta-analysis using the command “metaprop” was used to calculate the pooled prevalence of COVID-19 reinfection and 95% confidence interval (CI). To assess the sources and contribution of numerous factors to heterogeneity, we conducted univariable meta-regression and subgroup analysis by region (country), type of study, and type of specimen. Throat and oropharyngeal swabs were considered equivalent in the analysis.

I^2^ was used to check for heterogeneity between studies, and r^2^ was reported to refer to the proportion of variance explained by the covariates. We performed a sensitivity analysis to explore the effect of individual studies on the pooled estimate by eliminating the studies one by one and checking if there was substantial variation in the pooled estimates. Funnel plots and Egger’s test were used to check for publication bias. To determine temporal variations in the magnitude and direction of the pooled association estimate, we also performed a random-effects cumulative meta-analysis. The studies were first organized according to the year of publication and then sequentially included in the analysis in chronological order. The pooled estimates were updated as each study was added.

## Results

### Study selection

**Fig. 1:**
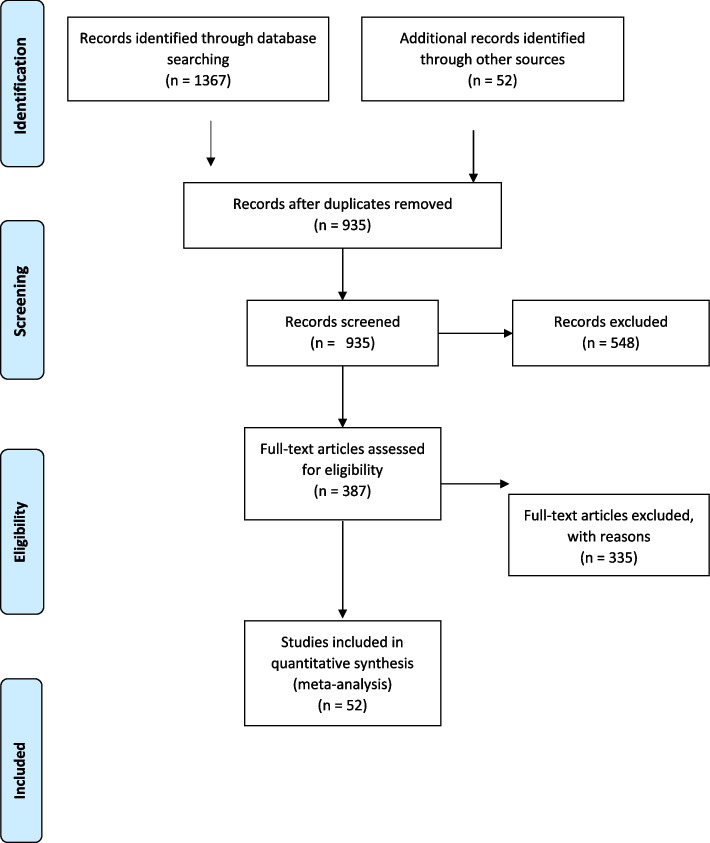
Flowchart

The search strategy and secondary bibliographic search yielded 1,419 relevant articles. After screening and removing duplicates, 52 studies with a total sample of 3,623,655 patients were finally included in the systematic review and meta-analysis (Fig. 1).

### Characteristics and quality of the studies included in the meta-analysis

Fifty-two studies included in this meta-analysis were published between 2019 and 2022, 24 (46.1%) were conducted in China. Thirty-three studies (63.5%) were cohort studies, and 19 (36.5%) were cross-sectional studies. We observed that cohort studies used data from active surveillance and testing while cross-sectional studies used data from routine laboratory testing.

The definition of COVID-19 reinfection differed from author to author; some authors considered reinfection as a new positive PCR following two consecutive negative PCR taken after primary infection [53–66]; some other studies considered reinfection as a new infection after 90 days of primary infection or after initial testing [29, 34, 36, 67–73]. Other studies considered reinfection as a new positive PCR 30–60 days after the first cured infection [74–78], and for others, the definition was retesting positive following prior complete recovery and/or after discharge [79–101].

Sixteen studies (30.8%) did not specify the type of samples used for PCR retesting. Among the studies that reported information on the type of sample, the most frequently used samples were nasopharyngeal swabs, which were solely used in 12 (23.1%) studies, and oropharyngeal samples, which were solely used in 8 studies (15.4%). Fifteen studies (28.8%) combined two or more samples (nasopharyngeal, oropharyngeal, sputum, and fecal samples), and nasal samples were used in only one study (1.9%) (Table 1).

**Table 1:** Characteristics of the studies included in the meta-analysis

Author name	Year of publication	Type of Study	Type of used data	Country	Definition of reinfection	Re-infection cases	Primary infection cases	Specimen
Ai Tang Xiao [53]	2020	Cross-sectional	Routine testing	China	Positive RT-PCR after two consecutive negative results	15	70	Throat swab
Anna Jeffery-Smith [102]	2021	Cohort	Active Surveillance / testing	England	Positive RT-PCR after at least 90 days after previous SARS-CoV-2 infection	10	656	Nose and throat swab
Anne Rivelli [34]	2022	Cohort	Active Surveillance / testing	USA	Positive PCR after 90 days of primary infection	159	2625	Nasopharyngeal, oropharyngeal, nasal
Bo Yuan [79]	2020	Cohort	Active Surveillance / testing	China	Retest positive after complete recovery	20	182	Nasopharyngeal and anal swab
Cheryl Cohen [68]	2022	Cohort	Active Surveillance / testing	South-Africa	Positive RT-PCR after at least 90 days after previous SARS-CoV-2 infection	87	749	Nasal swab
Christian Hansen [54]	2021	Cross-sectional	Routine testing	Denmark	Positive PCR test in two COVID-19 surge	72	11,068	Throat swab
Efren Murillo-Zamora [103]	2021	Cohort	Active Surveillance / testing	Mexique	Reappearance of symptoms at least 28 days after the initial confirmed infection	210	99,993	Not clearly specified
Emilie Finch [104]	2022	Cohort	Active Surveillance / testing	USA	Positive PCR test more than 30 days after initial seropositive result	14	309	Not clearly specified
Fabiánová K [105]	2021	Cross-sectional	Routine testing	Czech Republic	Positive RT-PCR after at least 90 days after negative PCR test	28	16,582	Not clearly specified
Fariba Zare [106]	2021	Cohort	Active Surveillance / testing	Iran	Positive PCR test more than 30 days after the first positive test	10	4039	Nasopharyngeal and pharyngeal swab
Flacco Maria Elena [36]	2021	Cohort	Active Surveillance / testing	Italy	Positive RT-PCR with at least 90 days, and with ≥ 2 consecutive negative tests between periods	24	7173	Nasopharyngeal swab
Flacco Maria Elena [107]	2022	Cohort	Active Surveillance / testing	Italy	2 Positive RT-PCR samples detected 45 days or more apart, with at least 1 intermediate negative PCR test	729	119,266	Nasopharyngeal swab
Guangming Ye [81]	2020	Cohort study	Active Surveillance / testing	China	Positive swab sample after recovery/discharge	5	55	Not clearly specified
Godwin E. Akpan [108]	2022	Cross-sectional	Routine testing	Liberia	Two positive RT-PCR samples detected 90 days or more apart, with at least 1 intermediate negative PCR test	13	5459	Not clearly specified
Hou-wei Du [109]	2020	Cohort	Active Surveillance / testing	China	Positive 2019 nCov nucleic acid test during follow-up after discharge	3	126	Nasopharyngeal and oropharyngeal swab
Hui Zhu [110]	2020	Cross-sectional	Routine testing	China	Positive RT-PCR after two consecutive negative results separated by at least 24 h	17	98	Nasopharyngeal swab or sputum
Ji Zhou [111]	2020	Cohort	Active Surveillance / testing	China	PCR during follow-up after prior SARS-Cov-2 infection	23	345	oropharyngeal swab
Jia Huang [112]	2020	Cohort	Active Surveillance / testing	China	Positive test after discharge	69	414	Nasopharyngeal swab
Jianghong An [56]	2020	Cohort	Active Surveillance / testing	China	Positive RT-PCR during follow-up after two consecutive negative results separated by at least 24 h	38	262	Nasopharyngeal and anal swab
Jie Chen [57]	2020	Cohort	Active Surveillance / testing	China	Positive RT-PCR during follow-up after two consecutive negative results separated by at least 24 h	81	1067	Throat swab
Jing Lu [113]	2020	Cross-sectional	Routine testing	China	Positive RT-PCR after two consecutive negative results separated by at least 24 h	87	619	Nasopharyngeal, throat and anal swab
Jing Yuan [62]	2020	Cohort	Active Surveillance / testing	China	Positive RT-PCR during follow-up after two consecutive negative results separated by at least 24 h	25	172	Nasopharyngeal and anal swab
Jinru Wu [114]	2020	Cross-sectional	Routine testing	China	Positive RT-PCR after discharge	10	60	Nasopharyngeal and anal swab
Ju Zhang [115]	2021	Cohort	Active Surveillance / testing	China	Positive SARS-Cov-2 test after negative PCR test between the two infection periods	6	273	Not clearly specified
Juliet R.C. Pulliam [116]	2022	Cross-sectional	Routine testing	South-Africa	Positive RT-PCR with at least 90 days after negative PCR test	105,353	2,942,248	Not clearly specified
Justin Wong [117]	2020	Cross-sectional	Routine testing	Brunei	Positive PCR during the study follow-up after 2 consecutive negative PCR at the discharge	21	106	Nasopharyngeal swab
Laith J. Abu Raddad [118]	2021	Cross-sectional	Routine testing	Qatar	After 45 days of the initial positive swab test	243	133,266	Nasopharyngeal and oropharyngeal swab
Lawandi A [71]	2022	Cohort	Active Surveillance / testing	USA	Positive RT-PCR with at least 90 days after negative qPCR test	253	131,773	Not clearly specified
Lei Pan [119]	2021	Cross-sectional	Routine testing	China	PCR redetected after discharge	20	1350	Throat or sputum swab
Leidi A [87]	2022	Cohort	Active Surveillance / testing	Switzerland	PCR during follow-up after prior SARS-Cov-2 infection	5	498	Nasopharyngeal and oropharyngeal swab
Maolu Tian [60]	2020	Cohort	Active Surveillance / testing	China	PCR during follow-up after two consecutive negative results	20	147	Oropharyngeal swab
Muhammad Syafiq Abdullah [88]	2020	Cohort	Active Surveillance / testing	Brunei	Retest positive after discharge	27	138	Nasopharyngeal and throat swab
Naila A Shaheen [89]	2022	Cohort	Active Surveillance / testing	Saudi Arabia	Positive RT-PCR tests during follow-up	132	35,288	Not clearly specified
Philippe Brouqui [72]	2021	Cohort	Active Surveillance / testing	France	Positive RT-PCR with at least 90 days after negative qPCR test	46	6771	Nasopharyngeal swab
Pilz Stefan [120]	2021	Cross-sectional	Routine testing	Austria	Positive SARS-Cov-2 tests after discharge	40	14,840	Not clearly specified
Rujun Hu [121]	2020	Cross-sectional	Routine testing	China	Recurrent positive RT-PCR after discharge	11	69	Nasopharyngeal swab
Salehi-Vaziri M [122]	2021	Cohort	Active Surveillance / testing	Iran	Positive rRT-PCR during the follow-up after the initial infection	5	1492	Not clearly specified
Sezanur Rahman [123]	2022	Cohort	Active Surveillance / testing	Bangladesh	Positive rRT-PCR during the follow-up after the initial infection	38	750	Nasopharyngeal swab
Sheehan M Meghan [73]	2021	Cohort	Active Surveillance / testing	USA	Positive RT-PCR with at least 90 days after previous SARS-CoV-2 infection	62	1278	not clearly specified
Shiua Luo [124]	2020	Cross-sectional	Routine testing	China	Symptoms and positive PCR tests after discharge	13	1673	Throat swab
Sivan Gazit [95]	2022	Cohort	Active Surveillance / testing	Israel	Positive PCR test during the follow-up period	1374	86,275	Not clearly specified
Tao Liu [58]	2020	Cross-sectional	Routine testing	China	Positive RT-PCR after two consecutive negative results separated by at least 24 h	11	150	Throat swab
Valeria Cento [125]	2020	Cohort	Active Surveillance / testing	Italy	Positive RT-PCR during follow-up after the discharge	264	2521	Nasopharyngeal swab
Wang Deng [65]	2020	Cross-sectional	Routine testing	China	Positive RT-PCR after the discharge	61	576	Nasopharyngeal and anal swab
Wang Xingyu [66]	2020	Cohort	Active Surveillance / testing	China	Positive SARS-Cov-2 test during the follow-up after the discharge	8	131	Not clearly specified
William R. Hartman [97]	2020	Cohort	Active Surveillance / testing	USA	Positive RT-PCR after 14 days of symptoms free	11	86	Nasopharyngeal swab
Yan Dong [126]	2021	Cross-sectional	Routine testing	China	Positive nucleic acid after the discharge	60	742	Nasopharyngeal swab
You Zou [59]	2020	Cohort	Active Surveillance / testing	China	Recurrent positive PCR after discharge with 2 consecutive negative tests	53	257	Throat swab
Youjiang Li [127]	2020	Cohort	Active Surveillance / testing	China	Positive PCR during follow-up after the discharge	4	13	Sputum, oral, nasal, and fecal swabs
Yun-Jung Kang [100]	2020	Cross-sectional	Routine testing	South Korea	Positive PCR after with discharge undetectable COVID-19 virus	292	8922	Not clearly specified
Zheng Jiazhen [101]	2020	Cohort	Active Surveillance / testing	China	Positive nucleic acid test during follow-up after discharge	27	285	Nasopharyngeal swab
Xiao Dong [78]	2021	Cross-sectional	Routine testing	USA	Positive RT-PCR with at least 60 days after 2 consecutive negative SARS-CoV-2 tests	23	690	Not clearly specified

All studies were conducted either in adults or in both adults and children, with the participants’ ages ranging from 0 to 100 years. No studies have considered vaccinated and unvaccinated participants separately.

The quality assessment results are presented in Table 2. Overall, 42 (80.8%) studies were of good quality, 10 (19.2%) were of fair quality, and no study was of poor quality.

**Table 2:** Quality assessment (NIH Quality assessment tool)

Author name	Q1	Q2	Q3	Q4	Q5	Q6	Q7	Q8	Q9	Q10	Q11	Q12	Q13	Q14	SCORE
Ai Tang Xiao	1	1	1	1	na	1	1	Na	1	1	1	na	1	1	11 Good
Anna Jeffery-Smith	1	1	1	1	na	1	1	na	1	1	1	na	1	1	11 Good
Bo Yuan	1	1	1	1	na	1	1	na	1	1	1	na	1	1	11 Good
Cheryl Cohen	1	1	1	1	na	1	1	na	1	1	1	na	1	1	11 Good
Christian Holm	1	1	1	1	na	1	1	na	1	1	1	na	1	1	11 Good
Efren Murillo-Zamora	1	1	1	1	na	1	1	na	1	1	1	na	1	1	11 Good
Emilie Finch	1	1	1	1	na	1	1	na	1	0	1	na	1	1	11 Good
Fabiánová K	1	1	1	1	na	1	1	na	1	1	1	na	1	1	11 Good
Fariba Zare	1	1	1	1	na	1	1	na	1	1	1	na	1	1	11 Good
Flacco Maria Elena	1	1	1	1	na	1	1	na	1	1	1	na	1	1	11 Good
Flacco Maria Elena	1	1	1	1	na	1	1	na	1	1	1	na	1	1	11 Good
Guangming Ye	1	1	1	1	na	1	1	na	1	1	1	na	1	1	11 Good
Godwin E. Akpan	1	1	1	1	na	1	1	na	1	1	1	na	1	1	11 Good
Hou-wei Du	1	1	1	1	na	1	1	na	1	1	1	na	1	1	11 Good
Hui Zhu	1	1	1	1	na	1	1	na	1	1	1	na	1	1	11 Good
Ji Zhou	1	1	1	1	na	1	1	na	1	1	1	na	1	1	11 Good
Jia Huang	1	1	1	1	na	1	1	Na	1	1	1	na	1	1	11 Good
Jianghong An	1	1	1	1	na	1	1	na	1	1	1	na	1	1	11 Good
Jie Chen	1	1	1	1	na	1	1	na	1	1	1	na	1	1	11 Good
Jing Lu	1	1	1	1	na	1	1	na	1	1	1	na	1	1	11 Good
Jing Yuan	1	1	1	1	na	1	1	na	1	1	1	na	1	1	11 Good
Jinru Wu	1	1	1	1	na	1	1	na	1	1	1	na	1	1	11 Good
Ju Zhang	0	0	1	0	na	1	1	na	1	1	1	na	1	1	8 Fair
Juliet R.C. Pulliam	0	0	1	1	na	1	1	na	1	1	1	na	1	1	9 Fair
Justin Wong	1	1	1	1	na	1	0	na	1	1	1	na	1	1	10 Fair
Laith J. Abu Raddad	1	1	1	1	na	1	1	1	1	0	1	na	1	1	11 Good
Lawandi A	1	1	1	1	na	1	1	na	1	1	1	na	1	1	11 Good
Lei Pan	1	1	1	1	na	1	1	1	1	0	1	na	1	1	11 Good
Leidi A	1	1	1	0	na	1	1	na	1	0	1	na	1	1	9 Fair
Maolu Tian	1	1	1	1	na	1	1	na	1	1	1	na	1	1	11 Good
Muhammad Syafiq Abdullah	0	1	1	1	na	1	0	na	1	1	1	na	1	1	9 Fair
Naila A Shaheen	1		1	1	na	1	1	na	1	1	1	na	1	1	11 Good
Philippe Brouqui	1	1	1	1	na	1	1	na	1	1	1	na	1	1	11 Good
Pilz Stefan	1	1	1	1	na	1	1	Na	1	1	1	na	1	1	10 Fair
Rujun Hu	1	1	1	1	na	1	0	na	1	1	1	na	1	1	10 Fair
Salehi-Vaziri M	1	1	1	1	na	1	1	na	1	1	1	na	1	1	11 Good
Sezanur Rahman	1	1	1	1	na	1	1	Na	1	1	1	na	1	0	10 Fair
Sheehan M Meghan	1	1	1	1	na	1	1	na	1	1	1	na	1	1	11 Good
Shiua Luo	0	1	1	1	na	1	0	na	1	1	1	na	1	1	9 Fair
Sivan Gazit	1	1	1	1	na	1	1	na	1	1	1	na	1	1	11 Good
Tao Liu	1	1	1	1	na	1	0	na	1	1	1	na	1	1	10 Fair
Valeria Cento	1	1	1	1	na	1	1	na	1	1	1	na	1	1	11 Good
Wang Deng	1	1	1	1	na	1	1	na	1	1	1	na	1	1	11 Good
Wang Xingyu	1	1	1	1	na	1	1	na	1	1	1	na	1	1	11 Good
William R. Hartman	1	1	1	1	na	1	1	na	1	1	1	na	1	1	11 Good
Yan Dong	1	1	1	1	na	1	1	na	1	1	1	na	1	1	11 Good
You Zou	1	1	1	1	na	1	1	na	1	1	1	na	1	1	11 Good
Youjiang Li	1	1	1	1	na	1	1	na	1	1	1	na	1	1	11 Good
Yun-Jung Kang	1	1	1	1	na	1	1	na	1	1	1	na	1	1	11 Good
Zheng Jiazhen	1	1	1	1	na	1	1	na	1	1	1	na	1	1	11 Good
Xiao Dong	1	1	1	1	na	1	1	na	1	1	1	na	1	1	11 Good
Anne Rivelli	1	1	1	1	na	1	1	na	1	1	1	na	1	1	11 Good

1: Yes

0: No

*na* not applicable

11–14: good quality

5–10: fair quality

0–4: poor quality

### Prevalence of COVID-19 reinfection

**Fig. 2:**
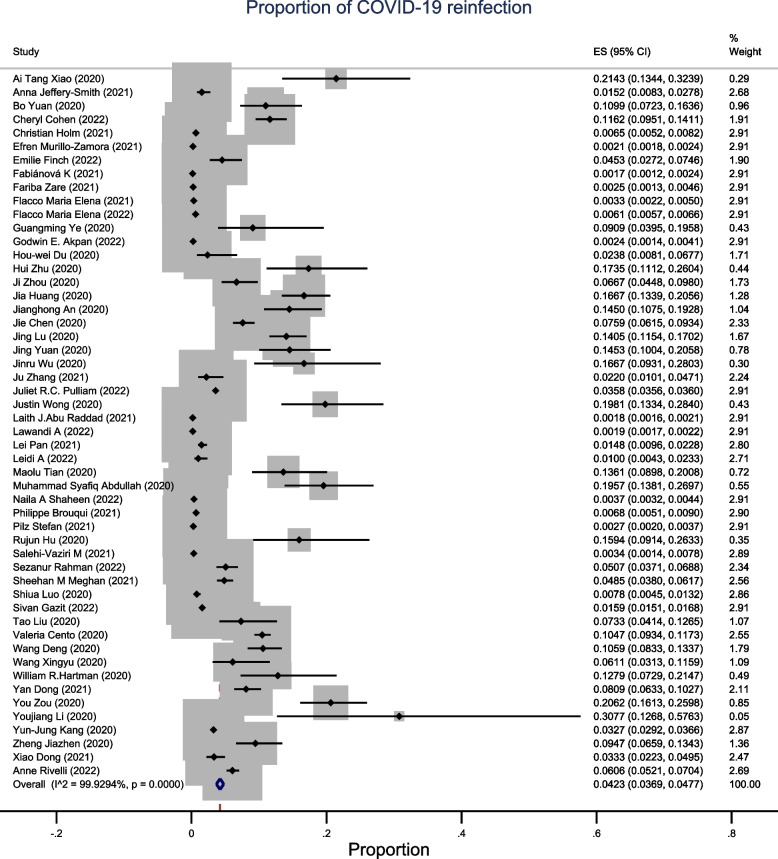
A meta-analysis of pooled estimates of recurrent COVID-19

**Fig. 3:**
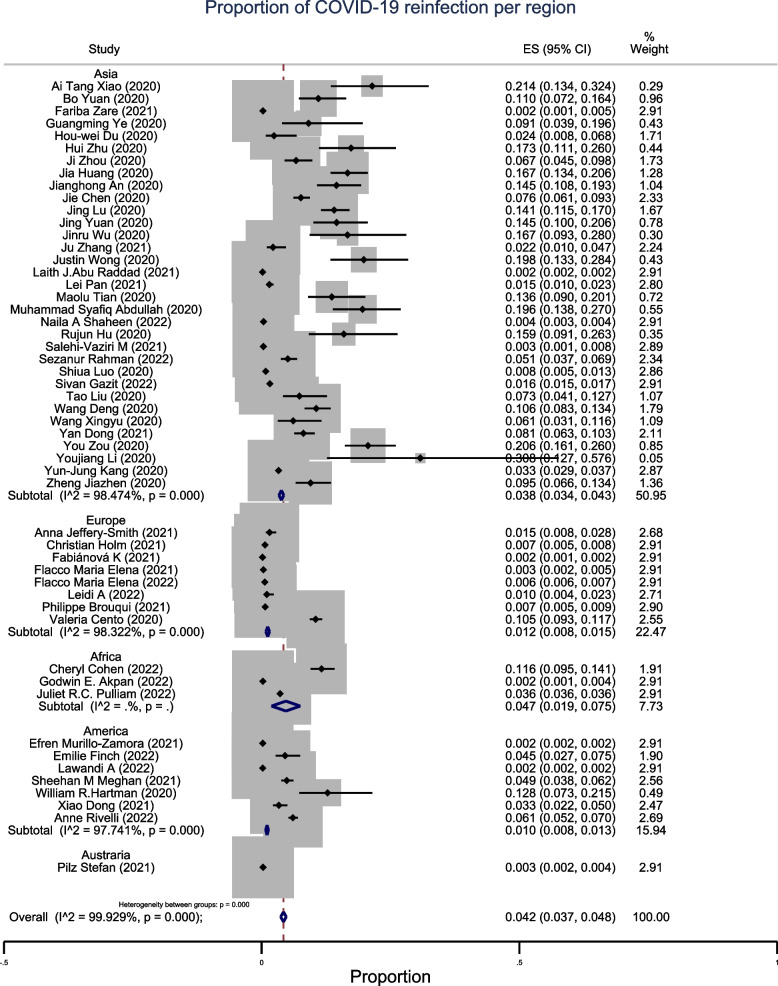
Sub-group meta-analysis by region of pooled estimates of recurrent COVID-19

**Fig. 4:**
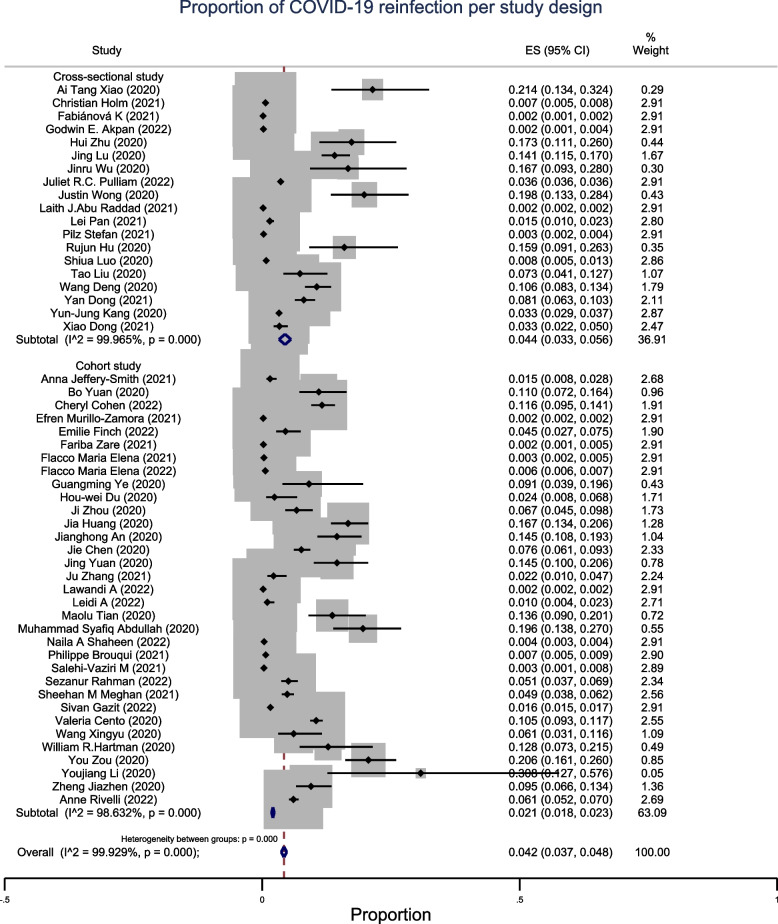
Sub-group meta-analysis by study design of pooled estimates of recurrent COVID-19

The pooled proportion of SARS-CoV reinfection was 4.2% (95% CI: 3.7–4.8%, *n* = 52), with high heterogeneity across continents (Fig. 2). The observed proportion of reinfection in studies conducted in Asia was 3.8% (95% CI: 3.4–4.3%; *n* = 33). In Europe, the proportion was 1.2% (95% CI: 0.8–1.5%; *n* = 8). The prevalence of reinfection was high in studies conducted in Africa, where the recorded proportion of reinfection was 4.7% (95% CI: 1.9–7.5%; *n* = 3). The proportion of reinfection in studies conducted in America was low at 1% (95% CI: 0.8–1.3%; *n* = 7) (Fig. 3). In the subgroup analysis by study design, cohort studies showed a proportion of SARS-CoV reinfection of 2.1% (95% CI: 1.8–2.3%; *n* = 33), below the overall pooled proportion of all studies. The proportion of reinfection recorded in the cross-sectional studies was 4.4% (95% CI: 3.3–5.6%; *n* = 19) (Fig. 4).

**Fig. 5:**
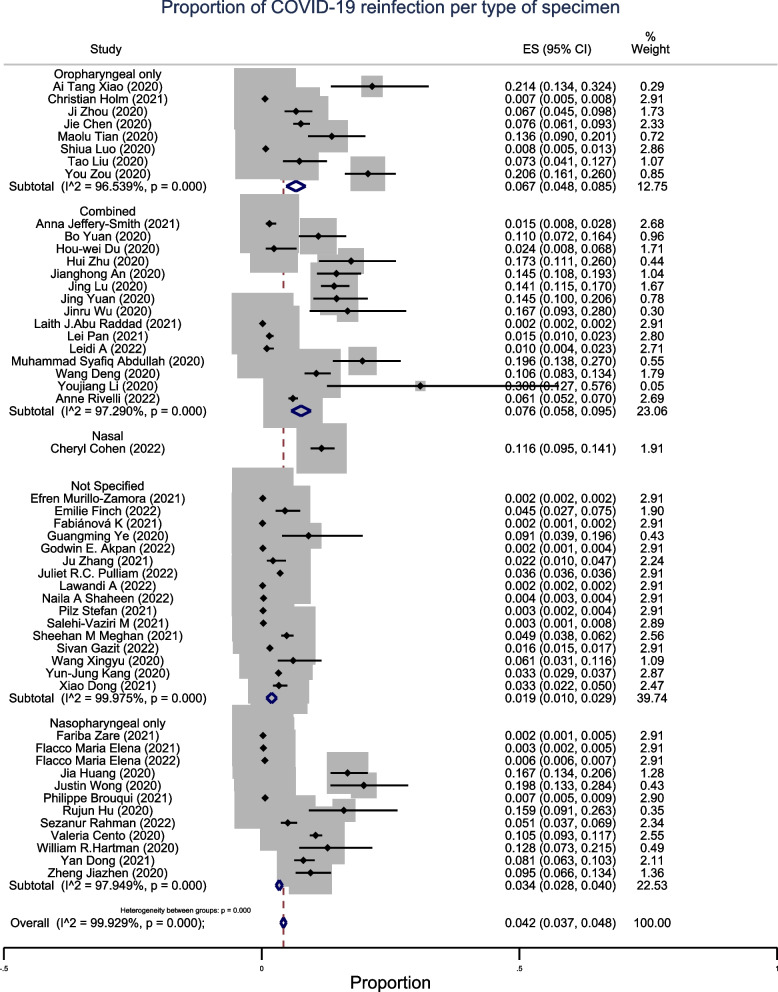
Sub-group meta-analysis by type of sample of pooled estimates of recurrent COVID-19

In the subgroup analysis by the type of specimen used for PCR retesting, we observed that the prevalence of reinfection of COVID-19 was 6.7% (95% CI: 4.8–8.5%; *n* = 8) in the studies that used oropharyngeal samples only. The prevalence was 3.4% (95% CI: 2.8–4.0%; *n* = 12) in studies that used nasopharyngeal samples only and 7.6% (95% CI: 5.8–9.5%; *n* = 16) in studies with combined samples. For studies that did not specify the type of sample used and those that used nasal swabs, the prevalence of COVID-19 reinfection was 1.9% (95% CI: 1.0–2.9%; *n* = 16) and 11.6% (95% CI: 9.5–14.1%; *n* = 1), respectively. (Fig. 5).

### Sensitivity analysis, cumulative effect assessment, and publication bias

**Fig. 6:**
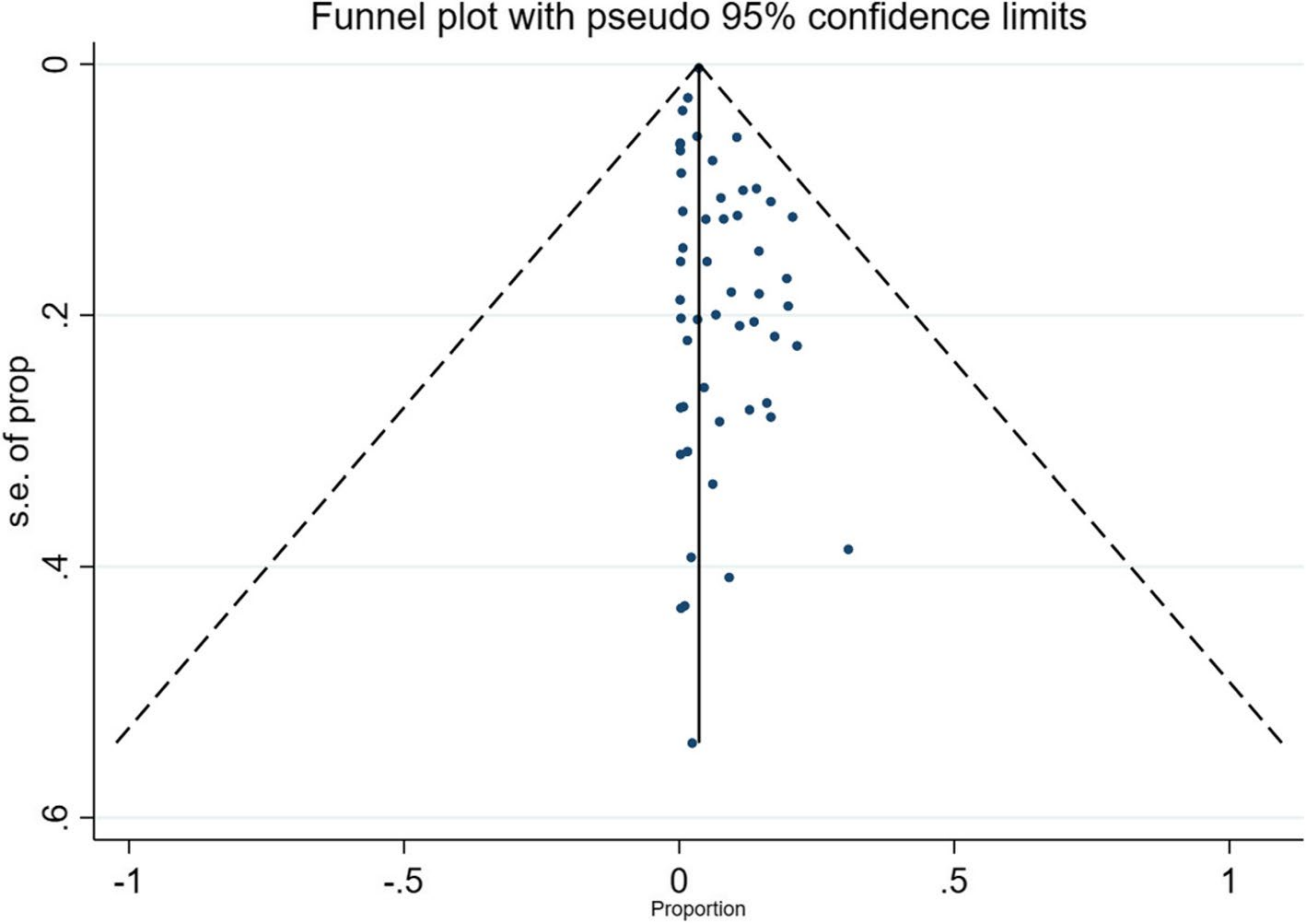
A funnel plot for publication bias

 +  + Sensitivity analysis was performed to evaluate whether an individual study had a predominant effect on the overall pooled prevalence of COVID-19 reinfection. This was performed by consecutively removing one study at a time while repeating the analysis. A study by Youjiang Li showed a substantial influence on the pooled prevalence, and its removal increased the prevalence to 13.1%. There was no discernible difference in the direction and magnitude of the pooled estimates across the years according to the cumulative random-effects meta-analysis. The funnel plot showed almost no asymmetry, and the Egger test for publication bias was not statistically significant (*P* = 0.27), suggesting no publication bias (Fig. 6).

## Discussion

This study determined the current prevalence of COVID-19 reinfection at global and regional levels. We found an overall proportion of COVID-19 reinfection of 4.2% (95% CI: 3.7–4.8%, *n* = 52).

Initially, it was debated whether it is possible to have COVID-19 reinfection [128]. The expert believed that infection with COVID-19 confers immunity, and that reinfection is less likely in previously infected people [129]. However, reinfection with COVID-19 is quite common, and naturally acquired immunity wanes within a few weeks of infection [129]. The reinfection was mostly observed with Omicron and its subvariants, which can evade natural immunity [129]. In a surveillance study conducted in South Africa, the Omicron variant was associated with an increased reinfection rate [29]. In contrast to other human coronaviruses, namely SARS-CoV, initially reported in Guangdong/China in 2002, and MERS-CoV, initially reported in Saudi Arabia in 2012, reinfection has not been reported as these infections were directly contained, leaving no time to document reinfection or recurrence [128]. However, reinfection was documented with other common coronaviruses within 4–48 weeks of primary infection (mean period of 37 weeks) [128, 130].

The prevalence of COVID-19 reinfection observed in this meta-analysis was low compared with that found in previous meta-analyses [131, 132]. This discrepancy in the prevalence of COVID-19 reinfection can be explained by these meta-analyses conducted early during the pandemic when few studies were available, in addition to the difference in the time of follow-up. A meta-analysis by Camilla et al. included 17 studies, and the follow-up period ranged from 1 to 60 days [131]. Another meta-analysis included 14 studies, and the median period from infection to recurrence ranged from 21 to 50 days [132]. This systematic review and meta-analysis included 52 relevant studies to determine the current prevalence of COVID-19 reinfection at the global and regional levels and considered a longer follow-up period.

There is a lack of a conventional definition of reinfection, and different authors of the studies included in existing meta-analyses have defined COVID-19 reinfection differently. Some authors have described cases of reinfection as continual viral shedding, whereas to ascertain reinfection, genomic sequencing should be performed to determine whether the second infecting virus is genetically different from the previous [51]. Zumrut et al. described a case of re-positivity of COVID-19 PCR after 27 days of a negative test, and it was inconclusive whether it was a reinfection or prolonged viral shedding, as no previous genotyping was performed for the first infection [133]. The CDC stated that viral shedding could be prolonged for up to 90 days, but even for severely infected persons, there was no replication-competent virus recovered after 10–20 days [134]; The ECDC proposed tests such as whole-genome sequencing and phylogenetic analysis to conclude on reinfection [48], and cases of genetically confirmed reinfection in a period of approximately 2 months from the previous infection have been reported in the literature [135, 136]. However, these genetic tests are not extensively available, and if considered widely, many cases of reinfection in many settings would not be recognized.

A higher proportion of recurrence of COVID-19 infection was observed in Africa (4.7%; 95% CI: 1.9–7.5%; *n* = 3) than that in other regions. This finding is unanticipated, as the incidence of COVID-19 in Africa is lower than that in other regions [17]. A review by Dufailu states that the incidence, hospitalization, and mortality rate of COVID-19 were lower in Africa than that in other continents [137].

This high rate of reinfection in Africa, where the pandemic was less severe, can be explained by the high population density in this region and the failure to implement pandemic control measures [137]. In addition, the low mortality rate observed in Africa, as well as the low incidence of severe cases of infection, should have led people to disregard restrictive measures and barrier actions against COVID-19, even in cases of previous infection, making reinfection an imminent occurrence [137, 138].

Other factors that should have influenced reinfection secondary to non-compliance with COVID-19 control measures by most of the population in Africa are the low vaccination rate, unsanitary conditions, lack of access to clean water, and lack of awareness [139]. A study by Wirsiy et al. published in 2020 mentioned that measures taken in Asia, Europe, and North America, such as physical (social) distance and consistent handwashing, were most difficult to implement in African countries where Internet connectivity was limited, population density, access to water was uneven, and social safety nets were limited [140].

On the other hand, this high prevalence observed in Africa is controversial, the study by Cohen et al., which has a high prevalence of COVID-19 reinfection used data from active surveillance and testing research compared to the other two African studies, which used data from routine testing. Active surveillance aims to detect every case. Cases are actively searched, tested, and followed—up, and their reporting is promoted; it provides the most complete, accurate, and timely information [141, 142] compared to passive surveillance, which relies on the analysis of data from reported cases and often lacks completeness [141, 142]. The difference between prevalence estimates obtained from routine laboratory testing vs active research surveillance data is less problematic in high-income countries, but particularly so in low- and middle-income countries, where testing capacity was correspondingly limited [143–146]. As gaps in testing were observed in Africa and more cases were missed [143–146], we would expect a difference between routine testing and active surveillance data. This, along with the relatively small number of studies from the continent, could have primarily raised the prevalence of COVID-19 reinfection, which is not necessarily real.

Oceania recorded the lowest prevalence of COVID-19 reinfection at 0.3% (95% CI: 0.2–0.4%; *n* = 1), but the sample size in this region was too small to predict the true estimates of reinfection.

Studies that used a combined type of specimens for PCR retesting had the highest prevalence of 7.6% (95% CI: 5.8–9.5%; *n* = 15). The studies that used nasopharyngeal specimens only had a lower prevalence of COVID-19 reinfection (3.4%; 95% CI: 3.7–4.8%; *n* = 12) compared with those that used only oropharyngeal specimens (6.7%; 95% CI: 4.8–8.5%; *n* = 8). These findings are the opposite of those observed in a meta-analysis by Azam et al., where the highest prevalence was found in studies that used a nasopharyngeal specimen only and the lowest in oropharyngeal specimens only [132].

These findings also disagree with those of a meta-analysis that assessed the positivity rate of COVID-19 using different types of specimens [147]. In the latter study, nasopharyngeal swabs showed a positivity rate of 45%, whereas that of oropharyngeal swabs was 7.6%, and the highest detection rate was found in bronchoalveolar fluids [147]. However, in agreement with the findings of this meta-analysis, other studies have shown that nasopharyngeal specimens yield a higher detection rate of COVID-19 than that of nasal or oropharyngeal swabs [148–151].

The discrepancy in these findings can be related to many studies considered in this meta-analysis that did not specify the type of specimen used for PCR retesting; however, this analysis considered throat and oropharyngeal swabs equivalent. Therefore, we hypothesize that combining two or more types of specimens should increase the detection rate of COVID-19, as substantiated by the findings of this study. However, more studies must validate this finding, and further meta-analyses will reduce this disagreement.

The difference between vaccinated and unvaccinated individuals in relation to the COVID-19 reinfection rate was not assessed because there was insufficient necessary information for this analysis. A prospective cohort study published in January 2022 by Sezanur Rahman revealed that new COVID-19 variants that emerged in 2021 could reinfect both naturally infected and vaccinated individuals and that being naturally infected confers better protection against COVID-19 for at least 6 months after primary infection [93]. Another systematic review indicated a risk of reinfection with COVID-19 in previously infected patients, including those vaccinated against the disease [152].

The severity of the reinfection was not addressed in this study. A systematic review conducted by Rubaid et al. in 2021 showed that the first infection and reinfection with COVID-19 showed a broadly similar pattern of clinical and management regimen but with a slightly higher severity among reinfected cases, evaluated by the need for mechanical ventilation and intensive care unit admission [153].

This meta-analysis has important implications for public health policies. The evidence of COVID-19 reinfection presented in this review should guide countries that demonstrate high reinfection prevalence to a future better preparedness for epidemic and pandemic diseases. Strategies should be set a priori on how preventive measures should be respected to prevent the spread of epidemics as well as reinfection.

The main strength of our study was the long period covered, which included studies from the first that reported reinfection in individuals pre-infected with COVID-19 until June 2022. This made it the first comprehensive meta-analysis to include many studies to date, which also considered the prevalence of COVID-19 reinfection by geographical area. In addition, most of the studies included in our analysis were of good quality.

The limitations of this study are that there is a difference in the definitions of reinfection used by the authors of the studies included in this meta-analysis, and it was difficult to compare findings across all studies. Additionally, no subgroup analysis was done by vaccination status or by definition of reinfection, and all sources of heterogeneity, such as the age group, were not explored because of a lack of appropriate related information among studies. Furthermore, the variability in sample size across the studies should have affected the prevalence and heterogeneity observed between studies.

## Conclusion

The current evidence shows that COVID-19 reinfection occurs and has a prevalence that varies worldwide, with the highest prevalence occurring in Africa. Therefore, preventive measures, including vaccination, should be emphasized to ensure control of the pandemic. More studies are needed to understand the rate of COVID-19 reinfection in consideration of different variants as well as comparing reinfection among vaccinated and unvaccinated persons. Factors that increase this risk of reinfection have not been well identified; hence, further studies can help in further clarification and future planning of preventive interventions to control this pandemic.

## Supplementary Information

**Additional file 1: Table S1.** Search strategy. **Table S2.** NIH Quality assessment tool questions.

## Data Availability

The authors declare that the data supporting the findings of this study are presented within the article and its supplementary materials.

## Abbreviations

CDC: Center for Disease Control
COVID-19: Coronavirus disease 2019
ECDC: European Center for Disease Control
MERS-CoV: Middle East Respiratory Syndrome coronavirus
NIH: National Institute for Health
PRISMA: Preferred Reporting Items for Systematic Reviews and Meta-Analyses
SARS-CoV: Severe acute respiratory syndrome coronavirus
WHO: World Health Organization
