# Supplementary material for: Global prevalence of coronavirus disease 2019 reinfection: a systematic review and meta-analysis
Source: BMC Public Health. 2023 Apr 28;23:778. doi: 10.1186/s12889-023-15626-7 (PMC10140730; doi:10.1186/s12889-023-15626-7)
Supplement: Supplementary file 1 — Additional file 1: Table S1. Search strategy. Table S2. NIH Quality assessment tool questions. [file 12889_2023_15626_MOESM1_ESM.docx]

**Appendix** Supplementary tables

**Table S1: Search strategy**

|  | COVID 19 **OR** SARS-CoV-2 Infection **OR** Infection, SARS-CoV-2 **OR** SARS CoV 2 Infection **OR** SARS-CoV-2 Infections **OR** 2019 Novel Coronavirus Disease **OR** 2019 Novel Coronavirus Infection **OR** 2019-nCoV Disease **OR** 2019 nCoV Disease **OR** 2019-nCoV Diseases **OR** Disease, 2019-nCoV **OR** COVID-19 Virus Infection **OR** COVID 19 Virus Infection **OR** COVID-19 Virus Infections **OR** Infection, COVID-19 Virus **OR** Virus Infection, COVID-19 **OR** Coronavirus Disease 2019 **OR** Disease 2019, Coronavirus **OR** Coronavirus Disease-19 **OR** Coronavirus Disease 19 **OR** Severe Acute Respiratory Syndrome Coronavirus 2 Infection **OR** SARS Coronavirus 2 Infection **OR** COVID-19 Virus Disease **OR** COVID 19 Virus Disease **OR** COVID-19 Virus Diseases **OR** Disease, COVID-19 Virus **OR** Virus Disease, COVID-19 **OR** 2019-nCoV Infection **OR** 2019 nCoV Infection **OR** 2019-nCoV Infections **OR** Infection, 2019-nCoV |
| --- | --- |
| AND | Reinfect* **OR** recurrent* **OR** Re-infection **OR** Re infection **OR** Re-infections **OR** Recurrent Infection **OR** Infection, Recurrent **OR** Recurrent Infections |

**Table S2: NIH Quality assessment tool questions**

| **Q1** | Was the research question or objective in this paper clearly stated? |
| --- | --- |
| **Q2** | Was the study population clearly specified and defined? |
| **Q3** | Was the participation rate of eligible persons at least 50%? |
| **Q4** | Were all the subjects selected or recruited from the same or similar populations (including the same time period)? Were inclusion and exclusion criteria for being in the study prespecified and applied uniformly to all participants? |
| **Q5** | Was a sample size justification, power description, or variance and effect estimates provided? |
| **Q6** | For the analyses in this paper, were the exposure(s) of interest measured prior to the outcome(s) being measured? |
| **Q7** | Was the timeframe sufficient so that one could reasonably expect to see an association between exposure and outcome if it existed? |
| **Q8** | For exposures that can vary in amount or level, did the study examine different levels of the exposure as related to the outcome (e.g., categories of exposure, or exposure measure as continuous variable)? |
| **Q9** | Were the exposure measures (independent variables) clearly defined, valid, reliable, and implemented consistently across all study participants? |
| **Q10** | Was the exposure(s) assessed more than once over time? |
| **Q11** | Were the outcome measures (dependent variables) clearly defined, valid, reliable, and implemented consistently across all study participants? |
| **Q12** | Were the outcome assessors blinded to the exposure status of participants? |
| **Q13** | Was loss to follow-up after baseline 20% or less? |
| **Q14** | Were key potential confounding variables measured and adjusted statistically for their impact on the relationship between exposure(s) and outcome(s)? |
